
JOURNAL INFORMATION
==============================
NLM Title Abbreviation: Wirtschaftsdienst
Journal ID: Wirtschaftsdienst
NLM Journal ID: 101086612

Wirtschaftsdienst (Hamburg, Germany : 1949)

ISSN: 0043-6275

ARTICLE INFORMATION
==============================
PMCID: PMC7335918
PMID: 32834155
DOI: 10.1007/s10273-020-2671-x
Article ID: 2671
Article version: 1
Subjects: Zeitgespräch

Das EU-Budget in der Corona-Krise

Henke Klaus-Dirk klaus-dirk.henke@tu-berlin.de

grid.6734.6 0000 0001 2292 8254 Institut für VWL und Wirtschaftsrecht, Fak. VII, TU Berlin, Fraunhoferstraße 33-36, 10587 Berlin, Deutschland
Electronic publication date: 2020 Jul 6
Print publication date: 2020
Volume: 100
Issue: 6
First page: 407
Last page: 410
Copyright: © Der/die Autor(en) 2020
License: Open Access Dieser Artikel wird unter der Creative Commons Namensnennung 4.0 International Lizenz veröffentlicht, welche die Nutzung, Vervielfältigung, Bearbeitung, Verbreitung und Wiedergabe in jeglichem Medium und Format erlaubt, sofern Sie den/die ursprünglichen Autor(en) und die Quelle ordnungsgemäß nennen, einen Link zur Creative Commons Lizenz beifügen und angeben, ob Änderungen vorgenommen wurden.
Die in diesem Artikel enthaltenen Bilder und sonstiges Drittmaterial unterliegen ebenfalls der genannten Creative Commons Lizenz, sofern sich aus der Abbildungslegende nichts anderes ergibt. Sofern das betreffende Material nicht unter der genannten Creative Commons Lizenz steht und die betreffende Handlung nicht nach gesetzlichen Vorschriften erlaubt ist, ist für die oben aufgeführten Weiterverwendungen des Materials die Einwilligung des jeweiligen Rechteinhabers einzuholen.
Weitere Details zur Lizenz entnehmen Sie bitte der Lizenzinformation auf http://creativecommons.org/licenses/by/4.0/deed.de.
License URL: https://creativecommons.org/licenses/by/4.0/

issue-copyright-statement: © The Author(s) 2020

==============================

Das Budget der Europäischen Union (EU) bedarf einer funktionalen Erneuerung; die Frage ist nur, wie sie aussehen soll. Eine Antwort ist nicht einfach, zumal der Sieben-Jahres-Haushalt des mittelfristigen Finanzrahmens nicht identisch mit den gesamten Finanzen der EU ist. Da es langer Ausarbeitungen bedürfte, um die europäische Finanzverfassung angemessen zu behandeln (Heinemann, 1998; Feld und Necker, 2010; Bundesministerium der Finanzen, 2016; Kielmansegg, 2020), sei angesichts des vorgegebenen Textumfangs nur eine wünschenswerte Entwicklung aufgezeigt.

## Aufgaben mit europaweitem Nutzerkreis

Mit dem Subsidiaritätsprinzip soll sichergestellt werden, dass Aufgaben nur dann auf die europäische Ebene gehören, wenn sie dort bestmöglich erfüllt werden. Bei strenger Auslegung dieses Prinzips durch den Sachverständigenrat zur Begutachtung der gesamtwirtschaftlichen Entwicklung (2016) wurden europaweite Güter und Leistungen in folgenden Bereichen vorgeschlagen (Europäische Kommission, 2017): Außenpolitik und Verteidigung, Migration und Asyl, Öffentliche Sicherheit und Strafverfolgung, Binnenmarkt, Wettbewerbs-, Klima- und Außenwirtschaftspolitik, Finanzmarktaufsicht sowie Kapitalmarktunion. Fiskal-, Arbeitsmarkt- und Sozialpolitik siedelt der Sachverständigenrat auf der nationalen Ebene an. Er bedauert die zunehmende Kompetenzaneignung der Europäischen Kommission und fordert eine stärkere Wahrung des Subsidiaritätsgedankens. Er greift den Vorschlag eines Subsidiaritätsgerichts mit rotierenden Richtern der höchsten Gerichte der Mitgliedstaaten auf und beklagt die ineffiziente Mittelverwendung der EU.

Ein Blick auf die Ausgabenseite des Haushalts 2020 zeigt, wie weit die Ausgaben von den zuvor genannten Aufgabenbereichen entfernt sind (vgl. Abbildung 1). Auch sieht man, dass die EU sicherlich die vorhandenen Mittel bei den nicht europaweiten öffentlichen Gütern (z.B. Agrar- und Strukturpolitik), die zusammen mehr als 70 % ausmachen, kürzen bzw. nach unten verlagern könnte (Bundesministerium der Finanzen, 2016, 21 ff.; Broer, 2017; Europäische Kommission, 2018).

## Corona-Krise als europaweite Herausforderung

Zu den traditionellen öffentlichen Gütern gehören beispielhaft das Klima oder der Wert der EU als „Friedensunion“. Wenn die öffentlichen Güter als Public Goods übersetzt werden, liegt es nahe, ihnen sprachlich die Public Bads gegenüberzustellen. Das Coronavirus fällt in diese Klassifikation der Güter, denn es ist ein europaweites öffentliches „bad“. Das Virus mit der damit verbundenen Unsicherheit trifft die gesamte Bevölkerung. Zu dieser Besonderheit gehören auch die Maßnahmen, mit denen die Mitgliedstaaten

- kollektiv das soziale Leben anhalten,
- massiv die Freiheitsrechte beschränken,
- die Wirtschaft bremsen und damit in eine tiefe Rezession steuern,
- gleichzeitig milliardenschwere Rettungspakete schnüren (Falk, 2020, 16).

Während die Pandemie durch SARS-CoV-2/COVID-19 ein öffentliches „bad“ sui generis ist, verändert der Staat gezielt die Präferenzen seiner Bürger, wenn er Public Bads mit Ge- und Verboten bekämpft. Auch die Anschnallpflicht beim Autofahren gehört hierher. Schwieriger wird es beim Impfschutz der Geimpften und seiner indirekten Wirkung auf nicht geimpfte Personen. Auch beim Tragen von einem

Abbildung 1
**EU-Haushalt 2020 – Mittel für Zahlungen**
in % der Gesamtausgaben und in Mrd. Euro

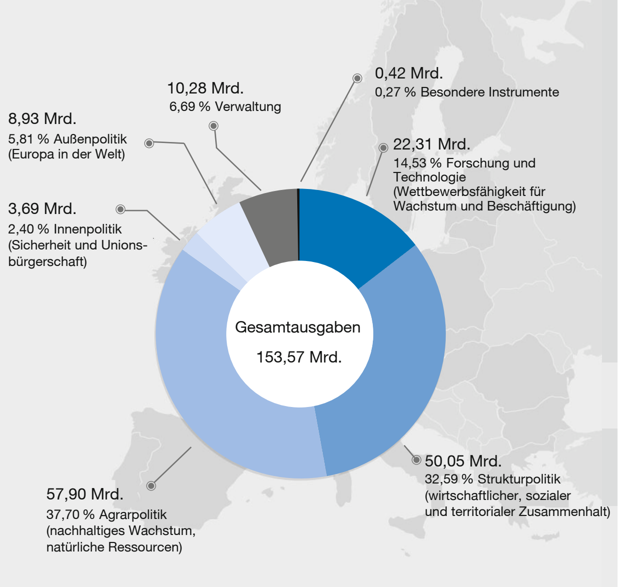

Quelle: Bundesministerium der Finanzen (2020b).

einfachen Mundschutz in Geschäften und in öffentlichen Verkehrsmitteln ergibt sich eine neue Situation, da der Schutz weit überwiegend beim Nichtträger liegt.

## Zur medizinischen Versorgung in der Corona-Krise

Angesichts der Pandemie mit ihrer globalen Wirkung auf die Mortalität und Morbidität stehen die Krankenversorgung und die gesundheitliche Betreuung der Bevölkerung im Vordergrund der Corona-Diskussion. Neue Medikamente und Impfstoffe gehören zu den Gütern und Leistungen, die sich in den öffentlichen Ausgaben niederschlagen. Unvorstellbar hoch wird der Betrag, wenn er nicht nur für rund 450 Mio. Menschen in der EU, sondern der gesamten Weltbevölkerung zur Verfügung gestellt werden könnte. Selbst wenn eine Impfpflicht in ihrem Umfang für alle hinterfragt werden kann, wird deutlich, wie stark die Unterschiede zwischen den Industrienationen und den ärmeren Ländern sind und dass die vorhandenen Ressourcen besser eingesetzt werden könnten (Schlette et al., 2007-2009).[1]

Die Verfügbarkeit und die Ausstattung der Krankenhäuser mit ihren Intensivstationen stehen auch zur Diskussion. Schon beim Stand der Krankenversorgung in der EU zeigt sich, dass die Mitgliedstaaten unterschiedlich gut auf diese Fälle vorbereitet waren – mit Deutschland und seiner ungewöhnlich guten Finanzlage als positivem Beispiel (Henke und Ettelt, 2020 und im internationalen Vergleich Schlette, 2020). Die Bundeskanzlerin fordert schon seit geraumer Zeit die Entwicklung leistungsfähiger europäischer Gesundheitssysteme, und die Gesundheitskommissarin der EU hat sich diesbezüglich mit Gesundheitsplänen der EU bereits zu Wort gemeldet (Kyriakides, 2020).

## Wirtschaftliche Implikationen der Corona-Krise

Die wirtschaftlichen Implikationen der COVID-19-Pandemie lassen sich in ihrem Ausmaß auf der nationalen Ebene der Mitgliedsländer europa- und weltweit noch gar nicht in all ihren Facetten erfassen. Die Wachstumsprognosen für die deutsche Wirtschaft zeigen zumindest kurzfristig stark sinkende Werte und die Umfragewerte zum Geschäftsklima waren noch nie so schlecht (Dorn et al., 2020). An der Bewältigung der wirtschaftlichen Implikationen mit ihren angebots- und nachfrageseitigen (Corona-)Schocks wird intensiv gearbeitet (Bofinger et al., 2020). In der aktuellen politischen Diskussion geht es dabei gleichzeitig um die unterschiedlichen Wege der EU, über ihren Haushalt oder andere Finanzierungsquellen zur Stabilisierung des Wachstums und der Beschäftigung in Europa solidarisch beizutragen. Die Bundesregierung bezifferte die Kosten der Corona-Krise für Bund und Länder bereits Mitte April 2020 auf 453 Mrd. Euro – für dieses Jahr. Hinzu kommen Staatsgarantien in Höhe von 820 Mrd. Euro. Der Bund wird deshalb 156 Mrd. Euro an neuen Krediten aufnehmen (Gatzke und Iser, 2020). Hinzugekommen sind noch die Ergebnisse der aktuellen Steuerschätzung für Deutschland mit erheblichen Ausfällen (Bundesministerium der Finanzen, 2020a).

Mitte 2020, spätestens mit der deutschen Präsidentschaft in der EU, wird es hier zu endgültigen wirtschaftspolitischen Lösungen kommen müssen. Dabei bedarf es der Zuordnung der Aufgaben und Ausgaben zum EU-Haushalt im Vergleich zu den anderen Finanzierungsträgern, also unter anderem der Europäischen Investitionsbank, dem Europäischen Stabilitätsmechanismus, der ECLL (Enhanced Credit Line Scheme)-Kreditlinie oder dem geplanten European Recovery Fund. Erschwert werden diese Berechnungen und Haushaltsverhandlungen dadurch, dass sich die Haushaltsperiode von 2021 bis 2027 erstreckt, der Brexit zu berücksichtigen ist und die unbekannten Langzeitwirkungen der Corona-Krise nicht zuverlässig antizipiert werden können. Schließlich ist zwischen den europaweiten und nationalen Corona-Hilfen zu unterscheiden. Dabei fällt auf, dass auf nationaler Ebene entsprechend der wirtschaftlichen Stärke der Länder natürlich unterschiedlich intensiv an der Eindämmung der Pandemie-Schäden gearbeitet wird. Um diese Ungleichheiten abzubauen, sind nach Aussagen der Vizekommissionspräsidentin Vestager im Rahmen der europäischen Solidarität erhebliche Zah-

1 Siehe auch Website des European Observatory on Health Systems and Policies (2016), z. B. Croatia.

lungen der EU vorgesehen (Der Spiegel, 2020; Frankfurter Allgemeine Zeitung, 2020).

Hinzu kommt, dass mit dem Budget der EU auch das Thema „Green Economy Platform" auf der Agenda steht. Sie umfasst die Handlungsfelder: Produktion und Ressourcen, Nachhaltigkeit und Finanzdienstleistungen, nachhaltige Energieversorgung, nachhaltiges Mobilitätssystem und die Zukunftsstadt (Bundesministerium für Bildung und Forschung, 2020). Bei der Verwendung der Mittel über den EU-Haushalt soll auf diese Belange Rücksicht genommen werden, sodass neben der Bekämpfung der Pandemie noch weitere Aufgaben anfallen.

## Einnahmenautonomie der EU

Bei der Finanzierung der EU geht es um Überlegungen, die mit der Corona-Krise (und dem Klimawandel) immer wichtiger werden. Auch wenn es derzeit weder eine Steuer- noch eine Verschuldungshoheit der EU gibt, gehören sie doch im Prinzip zu der wünschenswerten Autonomie eines öffentlichen Haushalts und damit auf die Agenda.

Was ist von einer „Steuer" als Anteil des Bruttonationaleinkommens (BNE) als „deklaratorische" Umsatz- oder Einkommensteuer (Henke, 2014 und Bundesministerium der Finanzen, 2016, 18 ff. und 24 ff.) und einer „ewigen Anleihe" (Soros, 2020) zu halten? Auf ihrer Grundlage ließen sich die europaweiten Aufgaben im Kontext der Corona-Hilfen und des wünschenswerten Klimawandels als öffentliche Güter finanzieren und die steigenden Ausgaben dauerhaft mit einem deutlich höheren EU-Budget bewältigen. Auf der Finanzierungsseite ist eine Art Besteuerung zumindest vorstellbar. Der größte Teil des EU-Budgets wird bereits heute über BNE-basierte Beiträge finanziert, sodass eine „Steuer"satzautonomie als ein Schritt in Richtung auf mehr Autonomie vorstellbar wäre, vergleichbar dem Solidaritätszuschlag. Sie erfasst die nationale Leistungsfähigkeit und erfordert keinen Harmonisierungsbedarf. Ähnlich sieht es bei der Verschuldung aus, die derzeit über die Europäische Investitionsbank (EIB) indirekt bereits läuft. Ob „ewige Anleihen", wie sie im angelsächsischen Bereich seit Jahrhunderten eingesetzt werden, eine europäische Zukunft aufweisen, muss geprüft werden. Sie scheitern bislang an den Römischen Verträgen von 1957 und werden im Kontext mit den süd- und südosteuropäischen Ländern diskutiert. Mit einer „ewigen Anleihe" und einer Art Steuer„befugnis" ließe sich die EU dauerhaft finanzieren. Die EU wird allerdings für einen „derartig mutigen Schritt auf absehbare Zeit nicht reif sein" (Richter, 2020; Müller und Richter, 2017).

## Die Rolle des EU-Budgets

Ein weiterer Blick auf den Haushalt der EU zeigt, dass die traditionellen Haushaltsgrundsätze bisher nur sehr unvollständig zu erkennen sind. Sie tragen dazu bei, dass vom Haushalt seine finanzwirtschaftliche, seine wirtschafts- und sozialpolitische sowie seine parlamentarische und administrative Lenkungsfunktion erfüllt werden (ausführlich: Zimmermann et al., 2017). Die Grundsätze der Klarheit, Vorherigkeit und vor allem der Einheit und Vollständigkeit gehören dazu. Die bestehende Fondswirtschaft mit ihren Nebenhaushalten verstößt gegen diese ebenso wie die Rabatte und Korrekturmechanismen. Die derzeitigen Bestandteile des europäischen Wiederaufbaus mit SURE (Support to mitigate Unemployment Risks in an Emergency), EIB, ESM (Europäischer Stabilitätsmechanismus), ECCL und der im Mai 2020 noch kontrovers diskutierte European Recovery Fund passen nicht zu der Forderung nach Einheit und Vollständigkeit des Budgets.

Vor allem aber geht es um mehr Transparenz in dem Zahlenwerk der EU-Finanzen und um seinen kaum erkennbaren europäischen Mehrwert mit den dazugehörigen Schwerpunkten. Hoffentlich zielen die in Brüssel Mitte 2020 laufenden Verhandlungen über den Haushalt mit seinem mehrjährigen Finanzrahmen (EU-Sondergipfel) wenigstens ein wenig in die aufgezeigte Richtung.

## Fazit

Sieht man abschließend den europäischen Einigungsprozess nicht nur als Verlängerung ihrer vielleicht in verschiedenen Zusammenhängen zu beklagenden Krisengeschichte und nimmt Gedanken von Graf Kielmansegg auf, dann könnte „an die Stelle eines unspezifischen, unumkehrbaren Integrationsprozesses ein projektgebundenes föderatives Handeln treten. Die Corona-Pandemie wäre dafür ein guter Anlass" (Kielmansegg, 2020; Enderlein, 2020). Auch Wolfgang Streeck (2020) sucht nach neuen Wegen für die EU. Mit seinem „Vorschlag einer begrenzten, nach gemeinsam ausgewählten Aufgabenfeldern gestalteten Europäischen Union" zeigt er in die gleiche Richtung. Vor diesem Hintergrund könnte die Corona-Krise tatsächlich einen Schub für ein stärkeres Europa auslösen, in dessen Mittelpunkt das EU-Budget steht (Merkel, 2020). Allerdings ist eine Stärkung des europäischen Parlaments dafür ebenso unabdingbar wie eine klare europäische Rechtsstaatlichkeit.[2]

2 Hingewiesen sei hier nur auf das jüngste Urteil des Bundesverfassungsgerichts zum Kaufprogramm der Europäischen Zentralbank (EZB) für Staatsanleihen, die Situation in Polen und Ungarn und die Asylpolitik mit der Flüchtlingsverteilung.

Prof. em. Dr. Klaus-Dirk Henke war Ordinarius für Volkswirtschaftslehre, insbesondere Finanzwissenschaft und Gesundheitsökonomie, an der Technischen Universität Berlin.


Literatur

Bofinger P Dullien S Felbermayr G Fuest C Hüther M Südekum J Weder di Mauro B Wirtschaftliche Implikationen der Corona-Krise und wirtschaftspolitische Maßnahmen Wirtschaftsdienst 2020 100 4 259 10.1007/s10273-020-2628-0 32336801 PMC7177746
Broer M Die EU und ihre Aufgaben, Ausgaben und Einnahmen — Theorie und Wirklichkeit ifo Schnelldienst 2017 70 6 6
Bundesministerium der Finanzen (2016), Reform der EU-Finanzierung: Subsidiarität und Transparenz stärken, Gutachten des Wissenschaftlichen Beirats beim BMF, Januar.
Bundesministerium der Finanzen (2020a), Pressemitteilungen, https://www.bundesfinanzministerium.de/Content/DE/Pressemitteilungen/Finanzpolitik/2020/05/2020-05-14-Steuerschaetzung.html (27. Mai 2020).
Bundesministerium der Finanzen (2020b), Infografiken, https://www.bundesfinanzministerium.de/Content/DE/Bilderstrecken/Infografiken/2019-11-19-einigung-eu-haushalt-2020/2019-11-19-eu-haushalt-2020-mittel-zahlungen.html (26. Mai 2020).
Bundesministerium für Bildung und Forschung (2020), Green Economy Plattform, https://www.green-economy-plattform.de/de/ (20. Mai 2020). Der Spiegel (2020), (Margrethe Vestager in einem Spiegel-Interview), Der Spiegel, 2. Mai, 78.
Dorn, F., C. Fuest, M. Göttert, C. Krolage, S. Lautenbacher, S. Link, A. Peichl, M. Reif, S. Sauer, M. Stöckli, K. Wohlrabe und T. Wollmershäuser (2020), Die volkswirtschaftlichen Kosten des Corona-Shutdown für Deutschland: Eine Szenarienrechnung, 22. März.
Enderlein, H. (2020), Jeder stirbt für sich allein, Der Spiegel, 11. April, 70 f. Europäische Kommission (2018), Legal texts and factsheets on the EU budget for the future, 2. Mai.
Europäische Kommission (2017), Weißbuch zur Zukunft Europas, 1. März, https://ec.europa.eu/commission/sites/beta-political/files/weiss-buch_zur_zukunft_europas_de.pdf (26. Mai 2020).
European Observatory on Health Systems and Policies (2006), Croatia
Health system review, Health Systems in Transition, 8(7), 200.
Falk, A. (2020), Das Empathie-Experiment, Frankfurter Allgemeine Zeitung, 11. Mai, 16.
Feld, L. P. und S. Necker (2010), Fiskalföderalismus in der Europäischen Union: Herausforderungen für die Reform der Finanzverfassung der EU. Frankfurter Allgemeine Zeitung (2020), (Margrethe Vestager in einem FAZ-Interview), Frankfurter Allgemeine Zeitung, 11. Mai, 17.
Gatzke, M. und J. C. Iser (2020), Kosten der Corona-Krise: Eine unbekannte mit sehr vielen Nullen, Die ZEIT, 15. Mai. Heinemann, F. (1998), EU-Finanzreform 1999.
Henke K-D EU-Haushalt: BNE-„Steuer“ zur Finanzierung? Wirtschaftsdienst 2014 94 3 156 10.1007/s10273-014-1651-4
Henke, K.-D. und S. Ettelt (2020), Coping with Covid-19, Health care capacity in Germany, Wall Street International Magazine, 18. April, https://wsimag.com/science-and-technology/61972-coping-with-covid-19 (20. Mai 2020).
Merkel, A. (2020), Deutsch-französische Initiative zur wirtschaftlichen Erholung Europas nach der Coronakrise, Presse- und Informationsamt der Bundesregierung, 18. Mai 2020.
Müller H Richter W F Europa am Scheideweg — ein Vorschlag zur politischen Weiterentwicklung Wirtschaftsdienst 2017 97 7 489 10.1007/s10273-017-2165-7
Kielmansegg, Graf P. (2020), Europa. Neu. Denken, Frankfurter Allgemeine Zeitung, 20. April.
Kyriakides, S. (2020), Gesundheitspläne der EU, Die Krebskrankheit hat meinen Blick auf das System geprägt, Gespräch, Der Tagessspiegel, 27. Januar.
Richter, W. F. (2020), Europäische Solidarität braucht ein echtes europäisches Parlament. Mit der geltenden europäischen Rechtsordnung lassen sich Corona-Bonds nicht begründen, Handelsblatt, 22. April, Gastkommentar.
Sachverständigenrat zur Begutachtung der gesamtwirtschaftlichen Entwicklung (2016), Jahresgutachten 2016/17, Tz 336–340.
Schlette, S. (2020), Germany’s response to the coronavirus pandemic, https://www.cambridge.org/core/blog/2020/04/08/germanys-response-to-the-coronavirus-pandemic/ (20. Mai 2020).
Schlette, S., K. Blum und R. Busse (Hrsg.) (2007–2009), Gesundheitspolitik in Industrieländern, Bände 7/8, 9, 10, 11, Bertelsmann Stiftung.
Soros, G. (2020), Die EU muss sich zusammenraufen — oder sie zerfällt, Der Spiegel, https://www.spiegel.de/wirtschaft/soziales/george-soros-fordert-ewige-anleihen-die-eu-muss-sich-zusammenraufen-a-6df11c82-a27f-42e6-b2db-14125c124efe (27. Mai 2020).
Streeck, W. (2020), Die Zeitbombe ist der Zerfall Italiens, Frankfurter Allgemeine Zeitung, 6. Mai, N4.
Zimmermann, H., K.-D. Henke und M. Broer (2017), Finanzwissenschaft — Eine Einführung in die Staatsfinanzen, 12. Aufl., 215–222.
